# Slow Walking in Individuals with Chronic Post-Stroke Hemiparesis: Speed Mediated Effects of Gait Kinetics and Ankle Kinematics

**DOI:** 10.3390/brainsci11030365

**Published:** 2021-03-13

**Authors:** Jing Nong Liang, Kai-Yu Ho, Yun-Ju Lee, Corey Ackley, Kiley Aki, Joshua Arias, Jassie Trinh

**Affiliations:** 1 Department of Physical Therapy, University of Nevada, Las Vegas, NV 89154, USA; KaiYu.Ho@unlv.edu (K.-Y.H.); ackleyc3@unlv.nevada.edu (C.A.); kileyaki@gmail.com (K.A.); ariasj11@unlv.nevada.edu (J.A.); jgtrinh@gmail.com (J.T.); 2 Department of Industrial Engineering and Engineering Management, National Tsing-Hua University, Hsinchu 300, Taiwan; yunjulee@ie.nthu.edu.tw

**Keywords:** post-stroke hemiparesis, walking speed, gait, slow walking, ground reaction forces

## Abstract

Post-stroke rehabilitation often aims to increase walking speeds, as faster walking is associated with improved functional status and quality of life. However, for successful community ambulation, ability to modulate (increase and decrease) walking speeds is more important than walking continuously at constant speeds. Increasing paretic propulsive forces to increase walking speed has been extensively examined; however, little is known about the mechanics of slow walking post-stroke. The primary purpose of this study was to identify the effects of increased and decreased walking speeds on post-stroke kinetics and ankle kinematics. Fifteen individuals with chronic post-stroke hemiparesis and 15 non-neurologically impaired controls walked over an instrumented treadmill under: slow, self-selected, and fast walking speeds. We examined the peak propulsive forces, propulsive impulse, peak braking forces, braking impulse, and ankle kinematics under each condition. When walking at slow walking speeds, paretic limbs were unable to reduce braking impulse and peak propulsive force or modulate ankle kinematics. Impaired modulation of paretic gait kinetics during slow walking places people post-stroke at high risks for slip-related falls. These findings suggest the need for developing gait retraining paradigms for slow walking in individuals chronically post-stroke that target the ability of the paretic limb to modulate braking forces.

## 1. Introduction

Stroke is the leading cause of adult long-term disabilities [1]. Individuals with hemiparesis resulting from a stroke possess significant impairments in locomotor function, resulting in slow walking speeds, asymmetrical gait patterns and fall risks, which negatively impacts functional or mobility independence and safety [2,3,4]. Faster walking speed is associated with enhanced quality of life [5], thus improving walking speed has been an important goal in stroke rehabilitation.

Despite the significance of aiming to increase walking speed in people post-stroke, in the context of functional community ambulation, long durations of continuous steady-state comfortable speed walking behavior has been found to be less important and also occurs less frequently [6]. Rather, gait speed modulation (i.e., increasing and decreasing walking speed) and gait initiation/termination has been identified as important and more frequent functional tasks for successful community mobility. For example, walking is often combined with talking, or other attention demanding activities, resulting in slower walking speeds [7], or in the attempt to change directions during walking, when negotiating a turn, walking speeds would be decreased compared to walking in a straight line [8]. Additionally, when an individual walks past a stationary object, or when a moving object approaches the individual, walking speed has been observed to be decreased [9].

In non-neurologically impaired individuals, during steady state walking (constant speed conditions), gait is characterized by symmetric anterior-posterior ground reaction forces generated by the two legs. Walking speed is regulated by anterior-posterior ground reaction force impulses (i.e., propulsion and braking), where, with increases in walking speed, propulsive and braking impulses are observed to increase [10]. With decreases in walking speed, magnitude of anterior-posterior ground reaction forces, and joint angles decreased [11].

In individuals post-stroke, during steady state walking, asymmetry in anterior-posterior ground reaction forces generated have been reported, with less propulsive forces generated by the paretic limbs. Furthermore, the more severe the hemiparesis, the greater the asymmetry in the propulsive and braking impulses observed [12]. With respect to the ability of foot-force control, during a posturally-supported locomotor task, the stroke-impaired system has been reported to be capable of generating foot forces that are appropriately controlled in magnitude and direction [13]. The propulsive forces generated by the paretic leg have been reported to be predictive of walking speed [12], and, in fast treadmill walking, increased paretic propulsive forces have been observed [14], suggesting the limited but potential capabilities of the stroke-impaired system to modulate foot forces under controlled locomotor conditions. However, the kinetics during slower walking speeds have yet to be examined in people with chronic post-stroke hemiparesis. Reduction in walking speed to below comfortable walking speeds has been considered to be a relatively more complex task, where different locomotor and postural control strategies are adopted, compared to comfortable walking speeds [15,16,17], and thus individuals post-stroke may not exhibit similar speed mediated changes compared to fast walking over the treadmill. In an intact nervous system, when walking speeds are reduced from comfortable to very slow walking speeds, the system switches from locomotor towards greater postural muscular control [15]. Faster locomotor speeds are potentially associated with greater suppression of vestibular drive, thus a reduction in walking speed would be associated with reduced suppression of this destabilizing vestibular drive. In conjunction with reduced proprioceptive input when speeds are reduced, increased dynamic instability makes slow walking more complex [18,19,20].

The primary goal of this study was to identify the effects of increased (120% of self-selected) and decreased (80% of self-selected) walking speeds on kinetics and ankle kinematics changes in people with chronic post-stroke hemiparesis. We hypothesized that in individuals with post-stroke hemiparesis, during fast walking (120% of self-selected speed), paretic legs would exhibit increased speed-related changes in kinetics (i.e., increased peak propulsive forces, propulsive impulse, peak braking forces and braking impulses) and ankle kinematics, compared to self-selected walking speed, but these variables would remain unchanged during slow walking (80% of self-selected speed).

## 2. Materials and Methods

### 2.1. Participants

Fifteen individuals (age ± SD = 61.52 ± 8.78 years old) with chronic post-stroke hemiparesis (2.76 ± 1.76 years post-stroke), participated in this study. Individuals in the stroke-impaired group were recruited via word of mouth or flyers from local stroke support groups. Fifteen similarly aged non-neurologically impaired individuals (59.32 ± 10.52 years old) were recruited as controls (Table 1).

Individuals post-stroke were included only if they were able to walk on a motor driven treadmill independently without assistive devices, such as cane or ankle-foot orthosis. If the participant usually wears an ankle-foot orthosis, it was removed during data collection. Exclusion criteria included: cerebellar strokes, neurological conditions other than the stroke, orthopedic conditions affecting the legs, history of hip/knee replacement, or the inability to comprehend/follow verbal instructions. Participants signed an informed consent form prior to data collection. The study protocol was approved by the Institutional Review Board at the University of Nevada, Las Vegas.

### 2.2. Procedures

Each participant attended a single session of data collection. After obtaining signed informed consent, the medical history, anthropometric data (body weight and height), vitals (blood pressure, resting heart rate and oxygenation), and Lower Extremity Fugl–Meyer Assessment were collected to assess their functional status and ability to participate in the study safely.

The kinematic data were captured at 200 Hz using a 12-camera motion analysis system (Vicon, Oxford Metrics Ltd., Oxford, UK) and ground reaction force data were collected at a sampling rate of 2000 Hz using force plates instrumented in a dual-belt treadmill (Bertec Corp., Columbus, OH, USA). Before data collection, the same investigator placed the reflective markers on all participants to ensure consistency of marker placements. The detailed definitions of the anatomical markers used in this study can be found in our previous work [21]. In brief, we placed the anatomical markers on the 1st and 5th metatarsal heads, medial and lateral malleoli, medial and lateral femoral epicondyles, the joint space between L5 and S1, greater trochanters, iliac crests, and anterior superior iliac spines (ASIS). Additional tracking markers were attached on participant’s lateral thigh, lateral leg, and heel counters bilaterally. A standing calibration trial was first obtained to define the segmental coordinate systems and joint axes. After the calibration trial, we removed all of the anatomical markers, except for those at the iliac crests and L5–S1 junction. The tracking markers remained on the participant throughout the walking trials. During data collection, participants wore a safety body harness when they walked over the treadmill. The harness did not provide any body weight support, but acted as a safety mechanism that catches them in events of fall.

Each participant walked over the motor-driven instrumented treadmill under 3 different speeds: self-selected walking speed (SSWS), fast walking speed (FWS), and slow walking speed (SWS). Each participant started with their SSWS condition, followed by the FWS or SWS conditions. The order of SWS and FWS conditions was randomized for each participant to ensure that sequence effect was minimized. The SSWS condition preceded the other 2 conditions so that the SWS (80% of SSWS) and FWS (120% of SSWS) can be determined. For each speed condition, once steady speed was attained, 3 trials of 30 s were collected continuously.

### 2.3. Data Analysis

The detailed data analysis procedures were published in our previous work [21]. First, we labeled the reflective markers using Vicon Nexus software (Oxford Metric Ltd., Oxford, UK) and calculated the kinematics of the lower extremities using Visual 3D software (C-Motion, Rockville, MD, USA). The un-filtered ground reaction forces were normalized to the body mass of the participants. For each participant, 5 strides of each 30 s trial were analyzed. Any crossover steps during the walking trials detected by the force plates were excluded from data analyses to minimize errors. As such, a total of 15 strides were analyzed for each condition. The kinetics were analyzed during the stance phase of gait, which was defined as the duration when the foot was in contact with the ground and presented with a positive vertical ground reaction force. To allow comparisons across all participants and 3 conditions, the duration of stance was normalized and expressed as % of stance phase for each trial. The highest positive and negative horizontal ground reaction forces were defined as the peak propulsive and braking forces during the stance phase, respectively. The propulsive impulse was defined as the integral of propulsive force over the stance time interval (i.e., % stance phase). Similarly, the braking impulse was defined as the braking force-time (i.e., % stance phase) integral. The data of both paretic and non-paretic limbs in stroke-impaired individuals, and the data of the right limb (non-impaired limb) of the non-neurologically impaired individuals were compared statistically.

### 2.4. Statistical Analysis

All statistical analyses were performed with SPSS 25.0 statistical software (International Business Machines Corp., Armonk, NY, USA). Independent samples *t*-test was used to compare the different walking speeds between the 2 groups. A 3 (limb: non-impaired, non-paretic, paretic) × 3 (speed: SWS, SSWS, FWS) mixed factorial ANOVA was conducted on each outcome variable. Where a statistically significant interaction effect was observed, it was further broken down using one way ANOVA and post hoc simple main effect. In cases where Mauchley’s test of sphericity was violated, Greenhouse–Geisser correction was used. Significant main effects and the results of post hoc t-tests with Bonferroni corrections were reported if there were no significant interactions. *A priori* significance was set at *p* ≤ 0.05.

## 3. Results

### 3.1. Walking Speeds

We observed statistically significant differences between groups for each speed condition. For each of the three speed conditions, the non-impaired group walked faster than the stroke-impaired group: SWS condition (*t*(27) = 3.46, *p* < 0.01), SSWS condition (*t*(27) = 3.57, *p* < 0.01), FWS condition (*t*(27) = 3.63, *p* < 0.01). (Table 1).

### 3.2. Kinetics

#### 3.2.1. Peak Propulsive Force during Stance

**Figure 1:**
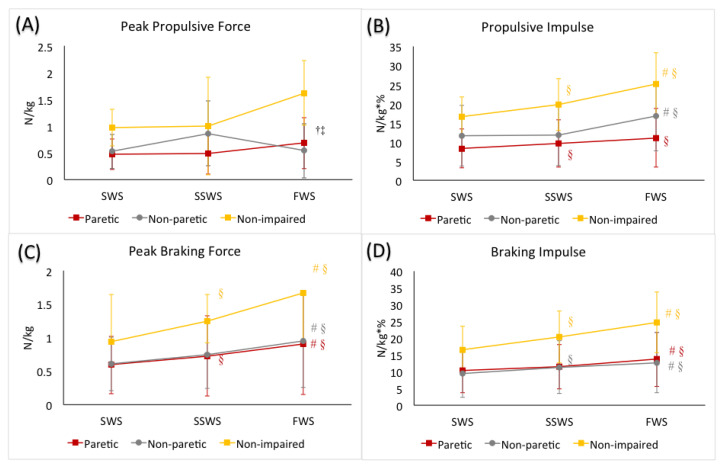
(**A**) peak propulsive force, (**B**) propulsive impulse, (**C**) peak braking force, and (**D**) braking impulse during the stance phase of gait during slow walking speed (SWS), self-selected walking speed (SSWS), and fast walking speed (FWS) conditions of the 3 limbs (paretic limb, non-paretic limb, and non-impaired limb). The error bars indicate the standard deviations. ^†^ denotes significant difference from the SSWS condition (*p* ≤ 0.05) when averaged across 3 limbs. ^‡^ denotes significant difference from the SWS condition (*p* ≤ 0.05) when averaged across 3 limbs. Colored-coded ^#^ denotes significant difference from the SSWS condition (*p* ≤ 0.05) for each limb. Colored-coded ^§^ denotes significant difference from the SWS condition (*p* ≤ 0.05) for each limb.

The mixed factorial ANOVA revealed no statistically significant interaction between speed and limb (F(1.27, 50.68) = 2.23, *p* = 0.11). We did observe a statistically significant main effect of speed (F(1.27, 50.68) = 14.70, *p* < 0.01). When averaged across all three limbs, peak propulsive forces were greater in the FWS condition compared to the SSWS (*p* < 0.01) and SWS (*p* < 0.01) conditions, respectively. We also observed a statistically significant main effect of limb (F(2, 40) = 8.44, *p* < 0.01). When averaged across walking speeds, the non-impaired limb generated greater peak propulsive forces than the non-paretic (*p* < 0.01) and the paretic (*p* < 0.01) limbs, respectively. (Table 2, Figure 1).

#### 3.2.2. Propulsive Impulse during Stance

The mixed factorial ANOVA revealed a statistically significant interaction between speed and limb (F(4, 80) = 3.93, *p* < 0.01). Post hoc analyses revealed, in the non-impaired limb, propulsive impulse generated was greater in the FWS condition (*p* < 0.01) and smaller in the SWS condition (*p* < 0.01), compared to the SSWS condition, respectively. In the non-paretic limb, propulsive impulse was greater in the FWS condition (*p* = 0.04) compared to the SSWS condition, but was not different between the SWS and SSWS conditions (*p* = 1.00). In the paretic limb, propulsive impulse generated was smaller in the SWS condition (*p* = 0.01) compared to the SSWS condition, but was not different between the FWS condition compared to the SSWS condition (*p* = 0.07). (Table 2, Figure 1).

#### 3.2.3. Peak Braking Force during Stance

The mixed factorial ANOVA revealed a statistically significant interaction between speed and limb (F(2.52, 50.41) = 6.65, *p* < 0.01). Post hoc analyses revealed that the non-impaired limb generated greater peak braking force in the FWS condition (*p* < 0.01) and smaller in the SWS (*p* > 0.01) condition, compared to the SSWS condition, respectively. Similar to the non-impaired limb, the non-paretic limb generated greater peak braking force in the FWS condition (*p* < 0.01) and smaller peak braking force in the SWS condition (*p* = 0.05) compared to the SSWS condition. Similarly, in the paretic limb, peaking braking force generated in the FWS condition was greater (*p* < 0.01), and peak braking force generated in the SWS condition was smaller (*p* < 0.01), compared to that in the SWSS condition, respectively. (Table 2, Figure 1).

#### 3.2.4. Braking Impulse during Stance

The mixed factorial ANOVA revealed a statistically significant interaction between speed and limb (F(4, 80) = 8.85, *p* < 0.01). Post hoc analyses revealed that the non-impaired limb generated greater braking impulse in the FWS condition (*p* < 0.01) and smaller in the SWS (*p* > 0.01) condition, compared to the SSWS condition, respectively. Similarly, in the non-paretic limb, greater braking impulse was observed in the FWS condition (*p* = 0.01) and smaller in the SWS (*p* = 0.01) condition, compared to the SSWS condition, respectively. However, in the paretic limb, greater braking impulse was observed in the FWS condition compared to the SSWS condition (*p* = 0.02), but braking impulse was comparable between SWS condition and SSWS conditions (*p* = 0.51). (Table 2, Figure 1).

### 3.3. Kinematics

#### 3.3.1. Ankle Angle at Initial Contact

**Figure 2:**
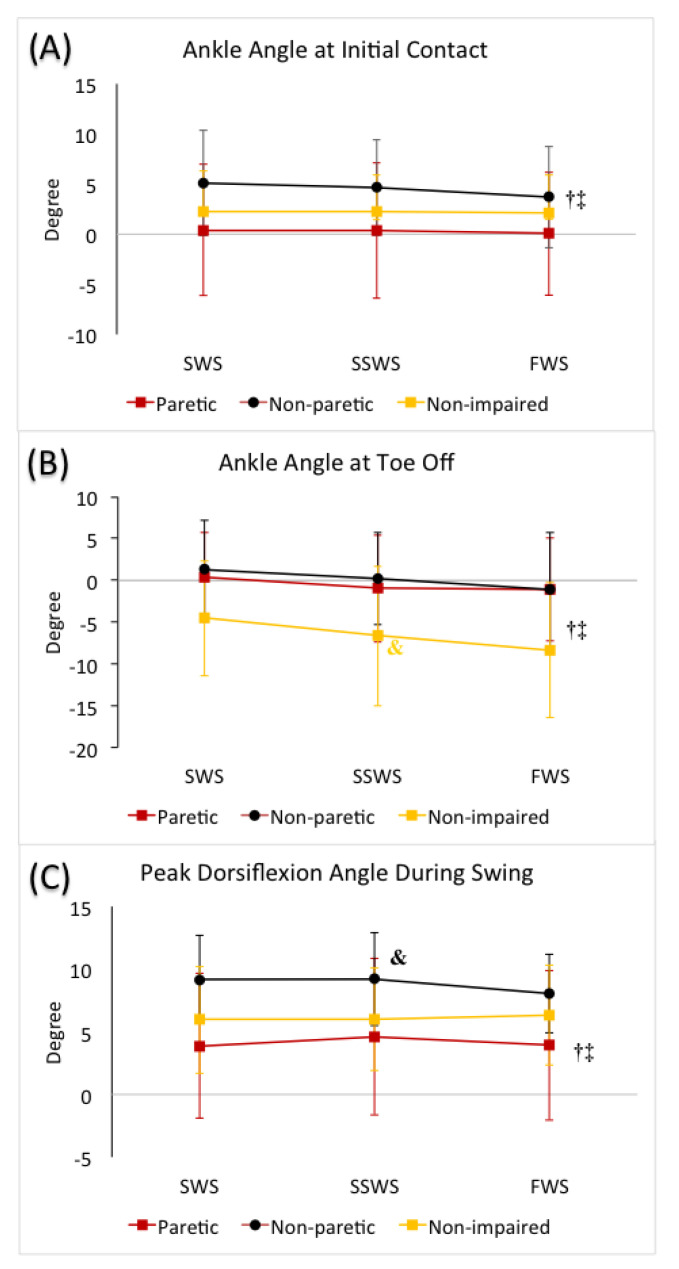
(**A**) Ankle angle at heel strike, (**B**) ankle angle at toe off, and (**C**) peak dorsiflexion angle during swing in slow walking speed (SWS), self-selected walking speed (SSWS), and fast walking speed (FWS) conditions across 3 limbs (paretic limb, non-paretic limb, and non-impaired limb). The error bars indicate the standard deviations. ^†^ denotes significant difference from the SSWS condition (*p* ≤ 0.05) when averaged across 3 limbs. ^‡^ denotes significant difference from the SWS condition (*p* ≤ 0.05) when averaged across 3 limbs. Color-coded ^&^ denotes significant difference from the paretic limb (*p* ≤ 0.05) for each limb when averaged across 3 speeds.

The mixed factorial ANOVA revealed no statistically significant interaction between speed and limb (F(4, 80) = 1.42, *p* = 0.23). We did observe a statistically significant main effect of speed (F(2, 80) = 4.81, *p* = 0.01). When averaged across the three limbs, we observed greater ankle dorsiflexion angle at initial contact during SWS condition compared to FWS condition (*p* = 0.03), whereas no difference was observed when comparing SWS (*p* = 1.00) or FWS (*p* = 0.09) conditions with SSWS condition, respectively. No statistically significant main effect of limb was observed (F(2, 40) = 2.64, *p* = 0.08) (Table 3, Figure 2).

#### 3.3.2. Ankle Angle at Toe off

The mixed factorial ANOVA revealed no statistically significant interaction between speed and limb (F(3.18, 63.67) = 0.90, *p* = 0.45). We did observe a statistically significant main effect of speed (F(1.59, 63.67) = 27.88, *p* < 0.01). When averaged across all three limbs, we observed greater plantarflexion at toe off in the FWS condition (*p* < 0.01), and smaller plantarflexion in the SWS condition (*p* < 0.01) compared to the SSWS condition. We also observed a statistically significant main effect of limb (F(2, 40) = 4.72, *p* = 0.01). When averaged across the different speeds, non-impaired limbs demonstrated greater plantarflexion compared to the non-paretic (*p* = 0.02) and paretic (*p* = 0.05) limbs, respectively, and there was no difference between the non-paretic and paretic limbs (*p* = 1.00) (Table 3, Figure 2).

#### 3.3.3. Peak Dorsiflexion during Swing

The mixed factorial ANOVA revealed no statistically significant interaction between speed and limb (F(4, 80) = 1.947, *p* = 0.11). No statistically significant main effect of speed was observed (F(2, 80) = 1.13, *p* = 0.33). We did observe a statistically significant main effect of limb (F(2, 40) = 4.97, *p* = 0.01). When averaged across the different speeds, greater dorsiflexion during swing was observed in the non-paretic limb compared to the paretic limb (*p* = 0.01) only. (Table 3, Figure 2).

## 4. Discussion

The primary aim of this study was to examine the ability of individuals with post-stroke hemiparesis to appropriately modulate their gait kinetics and ankle kinematics in response to increased (120% of self-selected) and decreased (80% of self-selected) walking speeds over a motor-driven treadmill. Our hypothesis that, in contrast to the non-neurologically impaired, the paretic gait kinetics and ankle kinematics when walking at slower walking speeds would remain unchanged was partially supported. We observed only reduced propulsive impulse and peak braking forces at slower speeds by the paretic limbs, while the others remain unchanged.

The non-impaired group walked at a faster SSWS compared to the stroke-impaired group, therefore both the SWS (80% of SSWS) and FWS (120% of SSWS) conditions were faster in the non-impaired group compared to the stroke-impaired group. Thus, on average across all speed conditions, the peak propulsive forces generated by the non-impaired limb was greater, accompanied by greater ankle plantarflexion at toe off, compared to the non-paretic and the paretic limbs.

With an increase in speed, the non-impaired limbs generated greater propulsive impulse, and with decrease in speed, propulsive impulse generated was reduced. This observed speed mediated modulation of propulsive impulse was in agreement with previous literature [10,11]. Contrary to the findings of Kesar et al. [14] where paretic propulsion was observed to be increased during fast walking, in the present study, while the paretic limbs were able to generate increased propulsive impulse with increasing speed from SWS to SSWS, any further increases in walking speed beyond SSWS was not accompanied by any change in paretic propulsive impulses. This suggests that at SSWS, the paretic limb already utilized its maximal capacity to generate propulsive impulse for achieving and maintaining that speed. Any further increases in paretic propulsive impulse generation to further increase walking speed was not possible. Thus, the ability of individuals post-stroke to achieve the FWS condition (120% SSWS) was likely the result of non-paretic limb contributions. The conflicting observation may potentially be due to the different functional status of the stroke participants between the two studies. On average, SSWS of the stroke participants in the previous study was 0.8 ± 0.2 m/s, and the fastest walking speed they were able to attain was 1.1 ± 0.3 m/s, which was significantly faster than our average SSWS and thus our FWS (0.57 ± 0.34 m/s). Due to the diverse nature of location and severity of stroke lesions with varying clinical presentations, it is critical to examine a large range of functional status to better understand the optimal threshold of deficits.

Similar to our observations for propulsive impulse, with increase in walking speed, the non-impaired limbs generated greater braking impulse, and with decrease in speed, the braking impulse decreased in magnitude. A comparable pattern was observed in the non-paretic limbs. In contrast, in the paretic limbs, while faster walking speed was accompanied by greater braking impulses generated, when slowing down the magnitude of braking impulses remained unchanged. This was in agreement with previous observations where the ability of the paretic limbs to appropriately control magnitude and foot-forces in the anterior-posterior direction during locomotion was examined using a controlled upright pedaling paradigm. When demands for postural loads have been alleviated, the stroke-impaired system was capable of generating foot forces that are appropriately controlled in magnitude and direction [13]. However, this appropriate foot-force control capability was disrupted under more challenging conditions, when individuals post-stroke have to simultaneously control for postural loads in addition to the locomotor task [22,23], comparable to overground/treadmill walking conditions. This inability to modulate braking impulse with reduction in speed can pose safety threats for individuals with chronic post-stroke hemiparesis. In order to successfully negotiate challenging walking environments in community ambulation, such as crossing the street before the light turns red, cluttered terrains, wet or slippery floors, walking past moving cars or persons, the ability to safely modulate walking speeds according to environment demands is critical. However, if a reduction in walking speed is accompanied by abnormally large braking impulses (overall larger force-time integrals) in the paretic limb, the patient will be at a higher risk for slip-related falls [24].

A limitation of the current study is the use of a motor-driven treadmill to control for participant walking speeds. While the use of an instrumented treadmill allowed us to collect continuous walking kinetics data, in contrast to overground walking using embedded force plates, treadmill walking induces a more consistent and symmetric gait pattern in people post-stroke [25], and the variability of the outcome measures is significantly reduced. This variability that was not captured can contain information that allows us to further understand the locomotor control of slow walking.

## 5. Conclusions

The inability of the stroke-impaired system to modulate gait kinetics while slowing down walking speed places individuals post-stroke at higher risks for slip-related falls. These findings provide evidence to support development of training paradigms targeting the ability of the paretic limbs to modulate braking forces relative to walking speeds, to be incorporated into gait re-training post-stroke.

## Figures and Tables

**Table 1:** Participant characteristics.

	Stroke-Impaired (*n* = 15)	Non-Neurologically Impaired (*n* = 15)
Age (mean ± SD, years)	61.52 ± 8.78	59.32 ± 10.52
Sex (number)	8 F/7 M	11 F/4 M
Slow walking speed (mean ± SD, m/s)	0.37 ± 0.23 ^†^	0.65 ± 0.21
Self-selected walking speed (mean ± SD, m/s)	0.47 ± 0.28 ^†^	0.92 ± 0.19
Fast walking speed (mean ± SD, m/s)	0.57 ± 0.34 ^†^	1.03 ± 0.35
Time post-stroke (mean ± SD, years)	2.76 ± 1.76	-
LE Fugl-Meyer score (/34)	25.80 ± 3.72	-
Paretic limb (number)	7 Left/8 Right	-

^†^ indicates statistically significant difference compared to non-neurologically impaired group (*p* < 0.01).

**Table 2:** Peak propulsive force, propulsive impulse, peak braking force, and braking impulse comparisons during the stance phase of gait between the slow walking speed (SWS), self-selected walking speed (SSWS), and fast walking speed (FWS) conditions of the 3 limbs (paretic limb, non-paretic limb, and non-impaired limb).

		Slow Walking Speed (SWS)	Self-Selected Walking Speed (SSWS)	Fast Walking Speed (FWS)
Peak Propulsive Force (*n*/Kg)	Paretic	0.48 ± 0.29	0.61 ± 0.40	0.71 ± 0.49
	Non-paretic	0.54 ± 0.32	0.68 ± 0.42	0.89 ± 0.50
	Non-impaired	0.97 ± 0.34	1.00 ± 0.91	1.61 ± 0.60
	*Averaged across 3 Limbs*	*0.66 ± 0.32*	*0.77 ± 0.64*	*1.09 ± 0.66* *^†,‡^*
Propulsive Impulse (*n*/Kg * %)	Paretic	8.53 ± 5.20	9.85 ± 6.28 ^§^	11.55 ± 7.83 ^§^
	Non-paretic	11.96 ± 7.69	12.34 ± 7.97	17.25 ± 8.90 ^#,§^
	Non-impaired	16.64 ± 5.10	19.81 ± 6.84 ^§^	25.15 ± 8.09 ^#,§^
Peak Braking Force (*n*/Kg)	Paretic	0.59 ± 0.33	0.72 ± 0.40 ^§^	0.91 ± 0.55 ^#,§^
	Non-paretic	0.60 ± 0.38	0.75 ± 0.50 ^§^	0.96 ± 0.67 ^#,§^
	Non-impaired	0.94 ± 0.43	1.25 ± 0.60 ^§^	1.66 ± 0.76 ^#,§^
Braking Impulse (*n*/Kg * %)	Paretic	10.37 ± 6.50	11.52 ± 6.68	13.83 ± 7.94 ^#,§^
	Non-paretic	9.53 ± 5.98	11.33 ± 7.30 ^§^	13.16 ± 8.46 ^#,§^
	Non-impaired	16.53 ± 6.98	20.31 ± 7.82 ^§^	24.72 ± 9.06 ^#,§^

^†^ denotes significant difference from the SSWS condition (* *p* ≤ 0.05) when collapsed across 3 limbs. ^‡^ denotes significant difference from the SWS condition (*p* ≤ 0.05) when collapsed across 3 limbs. ^#^ denotes significant difference from the SSWS condition (*p* ≤ 0.05) for each limb. ^§^ denotes significant difference from the SWS condition (*p* ≤ 0.05) for each limb.

**Table 3:** The comparisons of ankle kinematics between paretic, non-paretic, and non-impaired limbs across the 3 walking speeds.

		Slow Walking Speed (SWS)	Self-Selected Walking Speed (SSWS)	Fast Walking Speed (FWS)	*Averaged across 3 Speeds*
Ankle Angle at Initial Contact (°)	Paretic	0.5 ± 5.8	0.4 ± 6.8	0.0 ± 5.4	
	Non-paretic	5.0 ± 5.0	4.7 ± 4.8	3.6 ± 4.9	
	Non-impaired	2.3 ± 4.0	2.3 ± 3.6	2.1 ± 3.7	
	*Averaged across 3 Limbs*	*2.6 ± 5.2*	*2.4 ± 5.1*	*1.9 ± 4.8* *^†,‡^*	
Ankle Angle at Toe off (°)	Paretic	0.6 ± 4.7	−1.0 ± 5.9	−1.7 ± 5.9	*−0.7 ± 6.0*
	Non-paretic	1.3 ± 5.7	0.2 ± 5.5	−1.2 ± 6.5	*0.1 ± 6.1*
	Non-impaired	−4.5 ± 6.9	−6.7 ± 8.4	−8.4 ± 8.0	*−6.5 ± 7.8 ^&^*
	*Averaged across 3 Limbs*	*−0.9 ± 6.3*	*−2.6 ± 7.3* *^‡^*	*−3.9 ± 7.5* *^†,‡^*	
Peak Dorsiflexion during Swing (°)	Paretic	3.9 ± 4.7	4.6 ± 5.2	4.0 ± 5.0	*4.1 ± 6.0*
	Non-paretic	9.4 ± 3.5	9.2 ± 3.7	8.4 ± 3.3	*9.0 ± 3.5 ^&^*
	Non-impaired	6.0 ± 4.3	6.0 ± 4.1	6.3 ± 4.0	*6.1 ± 4.1*

Positive values indicate dorsiflexion and negative values indicate plantarflexion. ^†^ denotes significant difference from the SSWS condition (*p* ≤ 0.05) when averaged across 3 limbs. ^‡^ denotes significant difference from the SWS condition (*p* ≤ 0.05) when averaged across 3 limbs. ^&^ denotes significant difference from the paretic limb (*p* ≤ 0.05) when averaged across 3 speeds.
